# Inflammation-induced endothelial to mesenchymal transition promotes brain endothelial cell dysfunction and occurs during multiple sclerosis pathophysiology

**DOI:** 10.1038/s41419-018-1294-2

**Published:** 2019-01-18

**Authors:** Claudio Derada Troletti, Ruud D. Fontijn, Elizabeth Gowing, Marc Charabati, Bert van Het Hof, Imad Didouh, Susanne M. A. van der Pol, Dirk Geerts, Alexandre Prat, Jack van Horssen, Gijs Kooij, Helga E. de Vries

**Affiliations:** 1 Amsterdam UMC, Vrije Universiteit Amsterdam, Department of Molecular Cell Biology and Immunology, MS Center Amsterdam, Amsterdam Neuroscience, de Boelelaan 1117, Amsterdam, The Netherlands; 2 Neuroimmunology Research Laboratory, Centre de Recherche du Centre Hospitalier de l’Université de Montréal (CRCHUM), Montréal, Canada; 3 Department of Medical Biology, Amsterdam UMC, Amsterdam, The Netherlands

## Abstract

The blood-brain barrier (BBB) has a major role in maintaining brain homeostasis through the specialized function of brain endothelial cells (BECs). Inflammation of the BECs and loss of their neuroprotective properties is associated with several neurological disorders, including the chronic neuro-inflammatory disorder multiple sclerosis (MS). Yet, the underlying mechanisms of a defective BBB in MS remain largely unknown. Endothelial to mesenchymal transition (EndoMT) is a pathophysiological process in which endothelial cells lose their specialized function and de-differentiate into mesenchymal cells. This transition is characterized by an increase in EndoMT-related transcription factors (TFs), a downregulation of brain endothelial markers, and an upregulation of mesenchymal markers accompanied by morphological changes associated with cytoskeleton reorganization. Here, we postulate that EndoMT drives BEC de-differentiation, mediates inflammation-induced human BECs dysfunction, and may play a role in MS pathophysiology. We provide evidence that stimulation of human BECs with transforming growth factor (TGF)-β1 and interleukin (IL)-1β promotes EndoMT, a process in which the TF SNAI1, a master regulator of EndoMT, plays a crucial role. We demonstrate the involvement of TGF-β activated kinase 1 (TAK1) in EndoMT induction in BECs. Finally, immunohistochemical analysis revealed EndoMT-associated alterations in the brain vasculature of human post-mortem MS brain tissues. Taken together, our novel findings provide a better understanding of the molecular mechanisms underlying BECs dysfunction during MS pathology and can be used to develop new potential therapeutic strategies to restore BBB function.

## Introduction

The blood-brain barrier (BBB) is a selective and dynamic barrier that has a major role in maintaining brain homeostasis^1^. It is composed by specialized brain endothelial cells (BECs) which are tightly connected via adherent and tight junctions (AJs and TJs, respectively)^2,3^. Inflammation of the BECs and loss of their neuroprotective function is associated with several neurological disorders, including multiple sclerosis (MS). MS is a chronic autoimmune demyelinating disorder of the central nervous system (CNS), affecting more than 2 million people worldwide. Histopathological characteristics of MS include immune cell infiltration into the CNS, demyelination, glial cell activation and neurodegeneration^4,5^. We and others have shown that BBB dysfunction is an early key event in the pathogenesis of MS and may be considered as an attractive therapeutic target to fight neurological diseases^6–9^. The BBB gains its protective properties early in development, in a process called “barrierogenesis” where BECs become phenotypically and functionally specialized to fulfill the needs of the CNS tissue^10,11^. Particularly, endothelial junctions are important to create a tight barrier that restricts entry of unwanted and neurotoxic substances into the CNS. During adulthood, several signaling pathways are essential for the maintenance of the endothelium properties, including brain endothelium features^12,13^. Loss of these critical signaling events can result in endothelial cell death and dysfunction or it might results in a de-differentiation of the endothelial cells into mesenchymal cells in a process called endothelial to mesenchymal transition (EndoMT)^14–16^, a phenomenon similar to the better understood epithelial to mesenchymal transition^17^. EndoMT was first thought to be a purely developmental process, particularly during cardiac ontogeny^18^. However, recent reports indicated that EndoMT may also occur in adult tissues during different pathological disorders including brain diseases such as cerebral cavernous malformation, bacterial meningitis and brain tumors^19–21^. On a molecular level, EndoMT is characterized by the degradation of the endothelial vascular basement membrane, cell-to-cell junction rearrangements and reduced expression of functional endothelial markers. ECs undergoing EndoMT acquire mesenchymal and stem cells-like properties, including gain of migratory capacity, and increased expression of fibroblast and mesenchymal-specific markers, like fibroblast specific protein 1 (FSP1), fibronectin (FN1), and N-cadherin (CDH2)^14^. These phenotypic and functional changes require the interplay of different signaling pathways that activate transcription factors (TFs) from the SNAI, ZEB, and TWIST families^22^. Inflammatory mediators are known to activate different signaling pathways involved in EndoMT, including the NF-κB^23–25^ and the transforming growth factor (TGF)-β^16,17,26^. However, in contrast to its role in other tissues and diseases, the role of EndoMT in human BECs upon neuro-inflammation, as seen in MS, remains poorly understood. Here, we questioned whether human BECs undergo EndoMT upon inflammatory insult, thereby causing BECs dysfunction. Furthermore, analyzing human post-mortem brain tissue, we investigate whether EndoMT occurs during MS pathophysiology. We provide evidence that TGF-β1 and IL-1β drive EndoMT in human BECs and we highlight TAK1 as a central regulator of SNAI1, EndoMT and BBB dysfunction. Moreover, we observed vascular alterations associated with EndoMT in MS brain lesions, suggesting that EndoMT may represents a novel pathological mechanism underlying BBB dysfunction during MS pathophysiology.

## Materials and methods

### Cell cultures and treatment

The human BEC line hCMEC/D3 was kindly provided by Dr. Couraud^27^ (Institute Cochin, Université Paris Descartes, Paris, France). BECs were grown in EGM-2 Endothelial Cell Growth Medium-2 BulletKit, including basal medium and supplement components according to the manufacturer’s instructions (Lonza, Basel, Switzerland). Human embryonic kidney (HEK) 293T cells were cultured in Dulbecco’s modified Eagle’s medium supplemented with 10% fetal calf serum, 1% penicillin/streptomycin. Cell lines were grown in a 37 °C humidified atmosphere containing 5% CO_2_. To investigate inflammation-induced EndoMT, BECs were stimulated for 24 h with human recombinant TGF-β1 (10 ng/ml, R&D Systems, Minneapolis, MN, USA), human recombinant IL-1β (10 ng/ml, PeproTech, Rocky Hill, NJ, USA) or with a combination thereof, 1β in human endothelium serum-free medium (Invitrogen, Bleiswijk, The Netherlands). To analyze the nuclear protein content of NF-kB (p65 subunit) and SNAI1, BECs were treated for 4 h with the combination of TGF-β1 and IL-1β in human endothelium serum-free medium. To study the effect of 5Z-7-oxozeaenol (OZ), a potent and selective TGF-β activated kinase 1 (TAK1) inhibitor^28^, BECs were treated with OZ (1 µM) or with DMSO, as vehicle control, for 1 h prior to TGF-β1 and IL-1β treatment. To study the phosphorylation of TAK1, BECs were treated for 10 min with TGF-β1 and IL-1β together with the phosphatase inhibitor Calyculin A (20 nM, Cell Signaling Technology, Boston, MA, USA) in serum-free medium in the presence or absence of OZ. Primary human BECs were isolated, as previously described, from non-epileptic surgical human CNS material that was obtained from patients who underwent surgical treatment for intractable temporal lobe epilepsy^29–32^. Informed consent and ethic approval were given prior to surgery (ethical approval number BH07.001-BSP).

### Lentivirus-mediated overexpression (OE) of SNAI1

For the OE of SNAI1, we used the human SNAI1 lentiviral vector pLenti-GIII-CMV-GFP-2A-Puro (Applied Biological Materials, Viking Way Richmond, BC, Canada). Lentiviral particles were produced by co transfection of sub-confluent HEK293T cells with the SNAI1 plasmid or the pLenti-GIII-CMV-GFP-2A-Puro empty vector as a transfer vector with packaging plasmids (pMDLg/pRRE, pRSV Rev, pMD2.G)^33^ using calcium phosphate as transfection reagent. Infection-competent lentiviral particles were collected 48 h after transfection and the supernatant was centrifuged to remove cell debris. BECs were transduced with the SNAI1-expressing lentivirus. Forty-eight hours after transduction, SNAI1-expressing cells were selected by puromycin treatment (2 µg/ml).

### Lentivirus-mediated delivery of short-hairpin (sh)RNA

For knock-down (KD) of SNAI1, we used a vector-based shRNA delivery system. Plasmids from the TRC library (https://www.broadinstitute.org/rnai/trc/lib) targeting human SNAI1 were used to produce SNAI1 shRNA-expressing recombinant lentiviruses. Lentivirus was produced with SNAI1 shRNA plasmids as transfer vector as described above. BECs were transduced with shRNA-expressing lentivirus and selected as described above. The KD efficiency of all 5 constructs was tested, and the most effective construct used in subsequent experiments was TRC454083, encoding sequence GCAAATACTGCAACAAGGAAT that targets nucleotides 551–571 of the NM_005985.3 RefSeq. The TRC SHC002 vector containing an shRNA sequence that does not target any human genes (NTC) was used as a negative control.

### Electric cell-substrate impedance sensing (ECIS)

Primary human or cell line BECs (1 × 10^5^) were seeded on calf skin-derived collagen-coated 8W10+ECIS arrays (Ibidi, München, Germany) (collagen from Sigma-Aldrich, Saint Louis, MO, USA). Trans-endothelial electric resistance (TEER) of BECs was measured at multiple frequencies in real-time using an ECIS^TM^ Model 1600R (Applied BioPhysics, New York, USA). When maximum barrier resistance was reached, BECs were treated with OZ, TGF-β1 and IL-1β (either separately or in combination). The TEER of confluent, SNAI1-overexpressing BECs was monitored during barrier formation and compared to empty vector-transduced cells. All ECIS measurements were analyzed and subjected to mathematical modeling to calculate the barrier resistance (Rb) at each time point measured^34^.

### Permeability assay

BECs were cultured on calf skin-derived collagen-coated 0.4 µm pore size Transwell filters (Corning, Amsterdam, The Netherlands) (collagen from Sigma-Aldrich, Saint Louis, MO, USA). Paracellular permeability to FITC-dextran (70 kDa, Sigma Aldrich, Zwijndrecht, The Netherlands) in the luminal to abluminal direction was assessed in the presence or absence of OZ, TGF-β1, and IL-1β. FITC-dextran passing to the lower chamber was measured using a FLUOstar Galaxy microplate reader (BMG labtechnologies, Ortenberg, Germany).

### Quantitative reverse transcriptase PCR

RNA was isolated using the TRIzol® method (Life Technologies, Bleiswijk, The Netherlands) and cDNA was synthesized with the Reverse Transcription System kit (Promega, Leiden, the Netherlands). Sequences of all primers used are listed in Supplementary table 1. Oligonucleotides were synthesized by Invitrogen (Bleiswijk, The Netherlands). Quantitative Reverse Transcriptase PCR (qRT-PCR) reactions were performed using Fast SYBR Green Master Mix (Applied Biosystems, New York, NY, USA) on a ViiA 7 Real-Time PCR System Expression (Applied Biosystems, New York, NY, USA). Individual gene expression levels were normalized to GAPDH expression levels.

### Western blot and nuclear fractionation

BECs were washed with ice-cold phosphate-buffered saline (PBS) and lysed on ice with cell lysis buffer (Cell Signaling Technology, Boston, MA, USA) containing a protease and phosphatase inhibitor cocktail (Roche, Almere, The Netherlands, and Cell Signaling Technology, Boston, MA, USA, respectively), following the manufacturer’s instructions. Nuclear fractions were isolated using the NE-PER extraction kit (Thermo Fisher Scientific, Rockford, IL, USA), following the manufacturer’s guidelines. All samples were diluted in sample buffer (100 mM Tris-HCl pH 6.8, 4% SDS, 20% glycerol, 5% 2-mercaptoethanol and 2% bromophenol blue) and heated to 90 °C for 5 min. Lysates were separated on SDS-PAGE followed by transfer to nitrocellulose for immune-blot analysis. Blots were blocked for 1 h at room temperature with blocking buffer (LI-COR, Lincoln, NE, USA) or 5% milk in 0.1% Tween-20 solution. Subsequently, blots were incubated in blocking buffer containing 0.1% Tween-20 with antibodies against claudin-5 (CLDN5) (Thermo Fisher Scientific, Rockford, IL, USA), VE-cadherin (CDH5) (BD Biosciences, Franklin Lakes, NJ, USA), CDH2 (Sigma-Aldrich, Saint Louis, MO, USA), SNAI1 (Thermo Fisher Scientific, Rockford, IL, USA), β-actin (Santa Cruz, Dallas, TX, USA), α-tubulin (Clone DM1A, Cedarlane Laboratories, Burlington, Canada) and Lamin B (Santa Cruz, Dallas, TX, USA). Primary antibodies were detected and quantified by incubation with IRDye secondary antibodies (LI-COR) and use of the Odyssey infrared imaging system (LI-COR).

### Cell viability measurement

Confluent BECs monolayers were left untreated or treated with TGF-β1 and IL-1β for 48 h. Cell viability was determined using exclusion of trypan blue. The number of viable cells was divided by the total number of cells and plotted as % of live cells.

### Scratch assay

BECs were plated on a calf skin-derived collagen-coated 12-well plate and grown to confluency (collagen from Sigma-Aldrich, Saint Louis, MO, USA). Subsequently, a scratch was made with a p200 pipet tip. Debris was removed by washing the cells twice with serum-free medium and cells were treated with TGF-β1 and IL-1β or left untreated (control). After 24 h, cell migration was recorded using a Leica DM IL microscope (Leica, Mannheim, Germany). The migration rate was determined by measuring the cell-free area in captured images, using the ImageJ public domain software. Mathematical calculations were as previously described^35^. The migration rate was expressed as percentage of the scratched area covered by cells over time.

### Immunofluorescence microscopy

BECs were seeded in 8-well µ-slides (Ibidi, München, Germany). To investigate the occurrence of EndoMT upon pro-inflammatory stimulation and the nuclear accumulation of NF-kB (p65), cells were treated for 24 h and 4 h, respectively, with TGF-β1 and IL-1β. Where indicated, cells were pre-treated for 1 h with OZ (1 μM) or DMSO, as vehicle control. Cells were fixed with 4% formaldehyde or 75% ice-cold ethanol (Sigma-Aldrich, Saint Louis, MO, USA) and then permeabilized for 15 min using 0.1% Triton-X100 (Sigma-Aldrich, Saint Louis, MO, USA). Non-specific binding was blocked with 10% normal goat serum. Subsequently, cells were incubated with rabbit anti-CLDN5 (Thermo Fisher Scientific, Rockford, IL, USA), mouse anti-CDH5 (BD Bioscience, New Jersey, USA), mouse anti-occludin (OCLN) (Thermo Fisher Scientific, Rockford, IL, USA), mouse anti-CDH2 (Sigma-Aldrich, Saint Louis, MO, USA), rabbit anti-FSP1 (Abcam, Cambridge, UK) or mouse anti-NF-kB (p65 subunit) (Cell Signaling Technology, Boston, MA, USA). Primary antibodies were visualized using goat anti-mouse or goat anti-rabbit Alexa 488 (Molecular Probes, Eugene, OR, USA). To visualize F-actin, rhodamine-labeled Phalloidin (Thermo Fisher Scientific, Rockford, IL, USA) was used. Nuclei were visualized using DAPI (Molecular Probes). Stainings were recorded using the Nikon A1R+ confocal resonant scanning laser microscope (Nikon, Amsterdam, The Netherlands) or the Leica DMI 6000 SP8 microscope (Leica, Mannheim, Germany). NF-kB (p65 subunit) nuclear staining intensity was quantified using NIN ImageJ software analysis in three random fields in each well and data are presented as the average staining intensity of each treatment condition.

### Post-mortem human brain tissues

Brain tissue from 12 patients with clinically diagnosed and neuropathologically confirmed MS and from three control cases without neurological diseases was obtained after rapid autopsy and immediately frozen in liquid nitrogen (in collaboration with the Netherlands Brain Bank, Amsterdam). The Netherlands Brain Bank received permission to perform autopsies, for the use and for access to medical records for research purposes from the ethical committee of the VU university medical center Amsterdam, the Netherlands. White matter samples were selected on the basis of post-mortem magnetic resonance imaging. All patients and controls, or there next of kin, had given informed consent for autopsy and use of their brain tissue for research purposes. Clinical data are presented in Table 1.

**Table 1:** Patients details MS and non-neurological control patients details

Case	Gender	Age	Lesion	Type MS	PMD	Cause of death
07–275	Female	82	none	Control	05:10	Pneumonia by haemothorax
C14 P1E2	Male	64	none	Control	18	Unknown
C36 A1C2	Male	68	none	Control	30	Unknown
02–057 MRI1	Male	77	A	PP	04:15	Cerebrovascular accident
07–314 PLA14	Female	66	A	SP	6	Unknown
473 P2B3	Female	39	A	SP	9	Unknown
438 A1E5	Female	53	A	PP or SP	17	Unknown
11–093 PLA4	Male	56	CA	NA	10:10	Unknown
07–314 PLA13	Female	66	CA	SP	6	Unknown
2017–068 WML1	Female	48	CA	Unknown	09:20	Unnown
513 P2C5	NA	51	CA	SP	17	Unknown
04–247 MRI8	Male	70	CIA	Unknown	07:45	Cardiac arrest
07–127 PLA3	Female	48	CIA	NA	11:40	Unknown
99–067 PL11	Female	64	CIA	NA	07:45	Unknown
2017–068 WML2	Female	48	CIA	Unknown	09:20	Unnown

*PP* primary-progressive, *SP* secondary-progressive, *A* active, *CA* chronic active, *CIA* chronic inactive, *PMD* post-mortem delay, *NA* not applicable

### Immunohistochemistry

For immunohistochemical analysis, 5 µm cryosections of frozen brain tissues were fixed in ice-cold acetone for 10 min. After washing with PBS, sections were incubated overnight at 4 °C with primary antibodies against Myelin proteolipid protein (PLP) (Bio-Rad, Hercules, CA, USA), Human leukocyte antigen–antigen D related (LN3) (Thermo Fisher Scientific, Rockford, IL, USA), SNAI1 (Abcam, Cambridge, UK) or FSP1 (Abcam, Cambridge, UK). Subsequently, sections were washed with PBS and incubated with Envision Dual Link (DAKO, Glostrup, Denmark) for 30 min at room temperature, followed by visualization with the peroxidase substrate 3,3′-diaminobenzidine (DAKO, Glostrup, Denmark). Sections were incubated with hematoxylin for 1 min and extensively washed with tap water for 10 min. Finally, sections were dehydrated with ethanol followed by xylol and mounted with Entellan (Merck, Darmstadt, Germany). Protein expression was quantified using NIN ImageJ software analysis in three random fields in each slice, and data are presented as the average staining intensity of different groups.

Immunofluorescent double labeling was as following: after fixation in ice-cold acetone sections were permeabilized with 0.1% Triton X-100 (Sigma-Aldrich, Saint Louis, MO, USA), incubated for 30 min with 10% normal goat serum and finally incubated overnight at 4 °C with antibodies against SNAI1 (Abcam, Cambridge, UK) or FPS1 (Abcam, Cambridge, UK). The primary antibodies were visualized by incubation with goat anti-rabbit Alexa 488 (Molecular Probes) for 1 h at RT. Next, to visualize endothelial cells, sections were first incubated with anti-CD31 primary antibody (DAKO, Glostrup, Denmark) overnight at 4 °C which was visualized by incubation with goat anti-mouse Alexa-555 (Molecular Probes) for 1 h at RT. After washing, DAPI was used for nuclear staining and slides were mounted in Mowiol (Sigma-Aldrich, Saint Louis, MO, USA). Staining was recorded using a Nikon A1R+ confocal resonant scanning laser microscope (Nikon Amsterdam, The Netherlands).

### Statistical analysis

Results are shown as mean values with standard error of the mean (SEM). Statistical analysis was performed using GraphPad Prism software (v7 GraphPad Software, La Jolla, CA, USA). Comparisons between 2 experimental groups were made by unpaired Student *t*-test and between >2 groups by one-way ANOVA followed by post-hoc Bonferroni correction. Differences were considered significant at *p* < 0.05. All statistical tests are described in the figure legends.

## Results

### TGF-β1 and IL-1β induce human BECs dysfunction

**Fig. 1:**
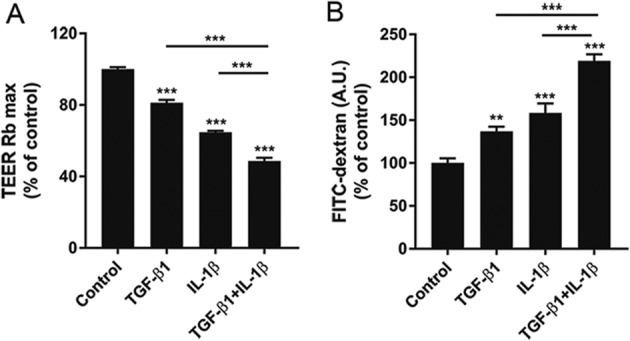
TGF-β1 and IL-1β induce human BECs dysfunction. **a**, **b** The effect of TGF-β1 and IL-1β on BBB function was assessed by measuring the TEER (Rb, barrier resistance) and the permeability of the BECs monolayers to FITC-dextran (A.U., arbitrary unit), both plotted as % of control. Data presented are the mean of triplicate values ± SEM of three independent experiments. Statistical analysis was performed using Student’s *t*-test where **p < 0.01, ***p < 0.001

To determine the effect of TGF-β1 and IL-1β on BECs function, we first analyzed barrier properties of the BECs upon stimulation with TGF-β1 and IL-1β as single stimuli. Next, because both TGF-β1 and IL-1β are secreted by reactive CNS-resident cells during MS pathogenesis^36–39^, we investigated the effect of the combined pro-inflammatory stimuli by measuring the TEER and the permeability to FITC-dextran. TGF-β1 and IL-1β stimulation resulted in a significant decrease in TEER (Fig. 1a) (18.7% and 35.2% reduction to control, respectively) and a profound increase in macromolecular permeability of the barrier (36.8% and 58.5% increase to control, respectively) (Fig. 1b). The combination of TGF-β1 and IL-1β was able to induce BECs dysfunction to a higher extent compared to the single treatments (51.3% TEER reduction and 119% permeability increase to control, respectively), suggesting an additive effect of IL-1β on TGF-β1 stimulation. Importantly, TGF-β1 and IL-1β did not appear to affect cell viability (Supplementary fig. 1). Taken together these data show a specific detrimental role for TGF-β1 and IL-1β on BECs barrier properties.

### Inflammation induces endothelial to mesenchymal transition of BECs

**Fig. 2:**
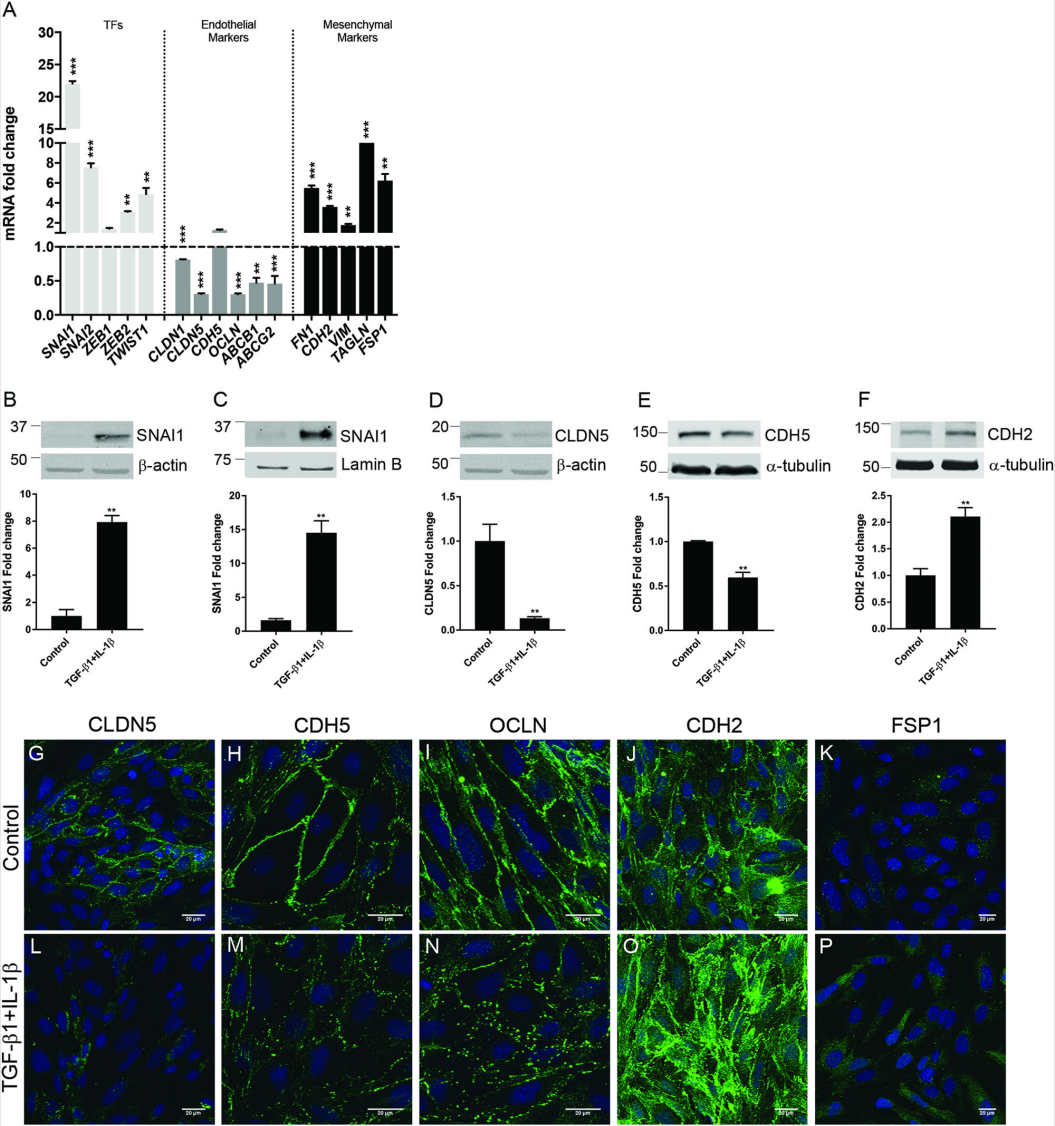
Inflammation induces EndoMT in BECs. **a** Confluent BECs were stimulated with TGF-β1 and IL-1β as described in the materials and method section, mRNA expression levels of different EndoMT-associated TFs, and endothelial or mesenchymal markers were determined by qRT-PCR. Values were normalized to *GAPDH* and plotted as fold change relative to control (dotted line). **b**–**f** TGF-β1- and IL-1β-stimulated BECs total and nuclear protein levels of SNAI1, CLDN5, CDH5, and CDH2 was assessed by western blot and expressed as fold change compared to control cells. Values were normalized to β-actin, α-tubulin or Lamin B levels. **g**–**p** CLDN5, CDH5, OCLN, CDH2, and FSP1 protein expression was assessed by immunofluorescence and imaged under the confocal microscope (scale bar 20 µm). Data presented are the mean of triplicate values ± SEM of three independent experiments. Statistical analysis was performed using Student’s *t*-test or one-way ANOVA, where *p < 0.05, **p < 0.01, ***p < 0.001 with post-hoc Bonferroni correction

TGF-β1 is a major inflammatory stimulus that promotes EndoMT^40^ and it has been reported that IL-1β amplifies its effect^41^. We hypothesized that TGF-β1-induced and IL-1β-induced BECs dysfunction might be due to BECs de-differentiation through EndoMT. To investigate this, we first tested whether TGF-β1 and IL-1β could activate SNAI1, a master regulator of EndoMT^22^. Interestingly, *SNAI1* mRNA levels were significantly induced only by TGF-β1, but could be further enhanced by IL-1β (Supplementary fig. 2A). We next assessed the capacity of TGF-β1 and IL-1β to induce EndoMT in BECs, by determining mRNA and protein levels of key molecules involved in this process. Our results indicated a significant upregulation of TFs associated with EndoMT, among which *SNAI1* showed the highest increase. Next to that, mRNA levels of several junctional markers, like claudin-1 and claudin-5 (*CLDN1, CLND5*), *OCLN*, ATP binding cassette subfamily B member 1 (*ABCB1*) and ATP-binding cassette sub-family G member 2 (*ABCG2*) were significantly reduced upon TGF-β1/IL-1β treatment. Finally, mRNA levels of key mesenchymal markers like *FN1*, *CDH2*, vimentin (*VIM*), transgelin (*TAGLN*) and *FSP1* were significantly upregulated upon TGF-β1/IL-1β stimulation (Fig. 2a). Treatment with TGF-β1 and IL-1β significantly upregulated nuclear and total SNAI1 (Fig. 2b, c). Moreover, a significant decrease in the AJ and TJ proteins like CLDN5 and CDH5, and an upregulation of mesenchymal markers CDH2 and FSP1 was observed upon TGF-β1/IL-1β stimulation at protein level, as shown by western blot and immunocytochemistry analysis (Fig. 2d–p). Furthermore, TGF-β1/IL-1β-treated BECs adopted a typical EndoMT spindle-like shape (Supplementary fig. 2B, C), revealed increased formation of actin stress fibers (Supplementary fig. 2D, E) and showed increased migration capacity compared to control BECs (Supplementary fig. 2F–J). Taken together, these data indicate that human BECs undergo EndoMT upon TGF-β1 and IL-1β stimulation. This transition is characterized by an increase in EndoMT-related TFs, a downregulation of brain endothelial markers, and an upregulation of mesenchymal markers accompanied by morphological changes associated with cytoskeleton reorganization.

### SNAI1 promotes EndoMT in BECs

**Fig. 3:**
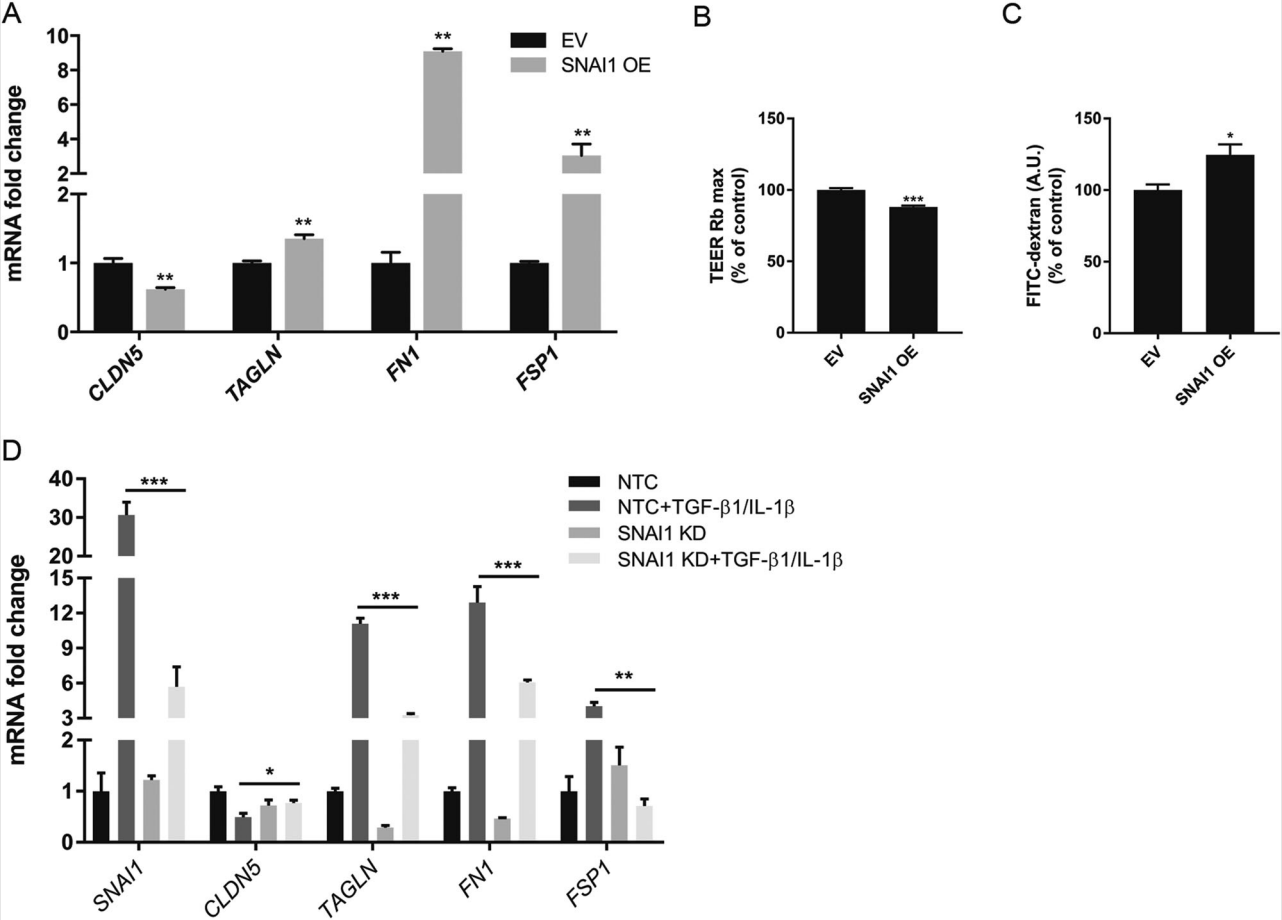
SNAI1 expression affects EndoMT in BECs. **a** *CLDN5*, *TAGLN*, *FN1*, and *FSP1* mRNA expression levels were measured in *SNAI1*-overexpressing cells by qRT-PCR. Values were normalized using *GAPDH* and plotted as fold change of control. **b**, **c** The TEER (Rb, barrier resistance) and the permeability to FITC-dextran (A.U., arbitrary unit) of the SNAI1-overexpressing cells were measured and plotted as % of control/EV-transduced BECs. **d** SNAI1 KD was achieved by lentiviral delivery of shRNA. *SNAI1*, *CLDN5, TAGLN, FN1* and *FSP1* mRNA expression level in SNAI1 KD cells were measured by qRT-PCR. Values were normalized using *GAPDH* and plotted as fold change relative to control. Data presented are the mean of triplicate values ± SEM of three independent experiments. Statistical analysis was performed using Student’s *t*-test or one-way ANOVA where *p < 0.05, **p < 0.01, ***p < 0.001 with post-hoc Bonferroni correction

To further characterize the role of SNAI1 during EndoMT in BECs, we generated a specific SNAI1-overexpressing cell line (Supplementary fig. 3). Resting SNAI1-overexpressing BECs displayed a phenotype closely resembling BECs treated with TGF-β1/IL-1β (Fig. 3a), indicating that SNAI overexpression was sufficient to induce an EndoMT phenotype in BECs. Moreover, SNAI1-overexpressing BECs displayed significantly reduced TEER (11.9% reduction) and increased permeability (24.6% increase) to fluorescent-labeled dextran in comparison to control/empty vector-transduced BECs (Fig. 3b, c). Finally, to corroborate the importance of SNAI1 in promoting TGF-β1/IL-1β-induced EndoMT, we reduced its expression by shRNA (Fig. 3d). Upon TGF-β1 and IL-1β stimulation, SNAI1-deficient BECs displayed significantly reduced *SNAI1* mRNA induction, a complete recovery of *CLDN5* mRNA expression and impaired induction of EndoMT, as marked by *FN1* mRNA expression levels (Fig. 3c) compared to NTC-transduced BECs. Taken together, these results suggest that SNAI1 expression is essential and sufficient to induce BEC de-differentiation, and promotes BECs dysfunction.

### TAK1 as central mediator of EndoMT

**Fig. 4:**
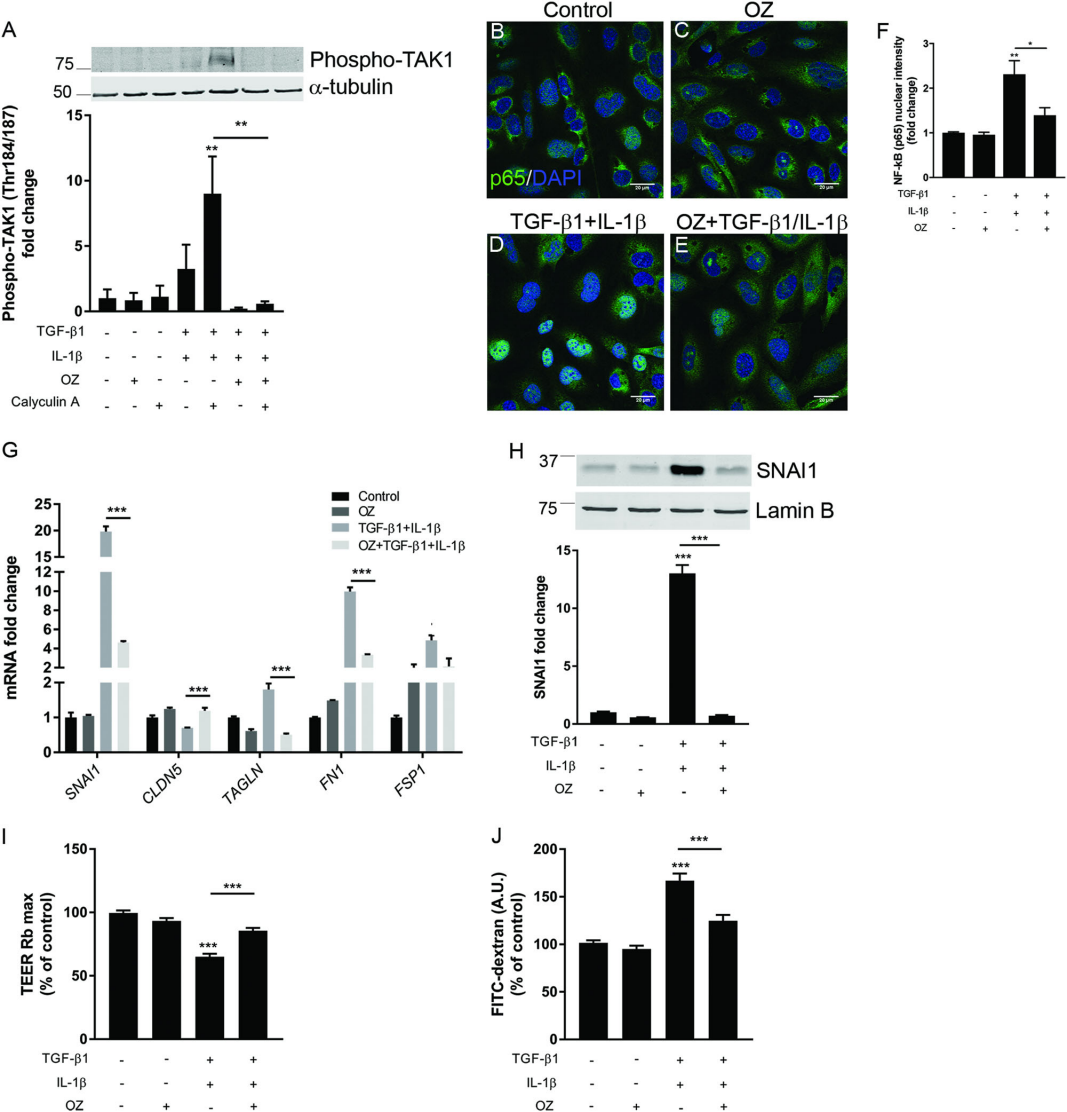
EndoMT can be reverted by inhibiting TAK1. **a** Confluent BECs were stimulated as indicated in the figure and as described in the materials and method section. Protein levels of phospho-TAK1 (Thr184/187) in cell lysates were determined by Western blot. Values were normalized to α-tubulin and plotted as fold change relative to control. **b**–**e** Stimulated-BECs were stained for NF-kB (p65 subunit) and analyzed using the A1R+ confocal resonant scanning laser microscope from Nikon (scale bar 20 µm). **f** NF-kB (p65 subunit) nuclear staining intensity was quantified using NIN ImageJ software analysis in three random fields in each well and is expressed as fold change intensity relative to unstimulated control cells. **g** mRNA expression levels of different EndoMT-related markers were determined by qRT-PCR upon stimulation of BECs. Values were normalized to *GAPDH* and plotted as fold change relative to control. **h** Nuclear protein content of SNAI1 was assessed by western blot and expressed as fold change compared to control cells. SnaI1 nuclear protein content was normalized using the nuclear envelope protein Lamin B. **i**, **j** The TEER (Rb, barrier resistance) and permeability to FITC-dextran (A.U. arbitrary unit) were measured and plotted as % of control BECs. Data presented are the mean of triplicate values ± SEM of three independent experiments. Statistical analysis was performed using one-way ANOVA where *p < 0.05, **p < 0.01, ***p < 0.001 with post-hoc Bonferroni correction

TAK1 is a serine/threonine protein kinase that has long been considered as a key mediator of inflammation and the non-canonical TGF-β and IL-1β signaling pathway^42,43^. TAK1 has recently been shown to play a role in the induction of EndoMT^44^. However, data are lacking on its role in EndoMT in BECs. We hypothesized that TAK1 might play a central role in TGF-β1- and IL-1β-induced EndoMT. In line with this hypothesis, our data showed a strong induction of TAK1 (Thr184/187) phosphorylation, a necessary step for its activation^45,46^, upon stimulation of BECs with TGF-β1 and IL-1β (Fig. 4a). Furthermore, we pharmacologically inhibited TAK1 activation using the selectively inhibitor OZ^28^. Pre-treatment of BECs with OZ resulted in an inhibition of TGF-β1 and IL-1β-induced TAK1 activation (Fig. 4a). Furthermore, to test the downstream effect of TAK1 inhibition, we analyzed the prototypical pro-inflammatory and pro-EndoMT transcription factor^25,47^ NF-kB (p65 subunit) nuclear content in BECs treated with OZ prior to TGF-β1 and IL-1β stimulation. We observed that TGF-β1 and IL-1β stimulation were able to increase the nuclear content of p65 (Fig. 4b–f). Next, our data showed that blocking TAK1 activation results in inhibition of the NF-kB signaling pathway, as marked by a significant reduction of the p65 nuclear content (Fig. 4d–f). Importantly, TAK1 inhibition partially reversed the EndoMT gene signature induced by TGF-β1 and IL-1β stimulation (Fig. 4g, h). Moreover, we studied the functional properties of the barrier endothelium upon TAK1 inhibition by pre-treating a BEC monolayer with OZ 1 h prior TGF-β1 and IL-1β stimulation. Inhibition of TAK1 resulted in a significant increase in TEER (Fig. 4i) (31.4% increase) and a decrease in macromolecular permeability (23.7% decrease) of the barrier compared to TGF-β1- and IL-1β-treated cells (Fig. 4J). Finally, we validated data obtained with the BEC cell line using primary human BECs. We first measured the TEER of primary human BECs in response to TGF-β1/IL-1β stimulation and to OZ pre-treatment. Our data indicate that primary human BECs respond to TGF-β1 and IL-1β, and that OZ pre-treatment restored the detrimental effect of TGF-β1/IL-1β stimulation, similar to the BEC cell line (Supplementary fig. 4A). Also, SNAI1 level regulation showed the same pattern as compared to BECs cell line (Supplementary fig. 4B). Collectively, our data demonstrate a central role for TAK1 in promoting SNAI1 transcription and, consequently, inducing EndoMT in BECs. Importantly, pharmacological inhibition of TAK1 activation by TGF-β1 and IL-1β prevents EndoMT and restores barrier properties.

### EndoMT occurs in the vasculature of MS patients

**Fig. 5:**
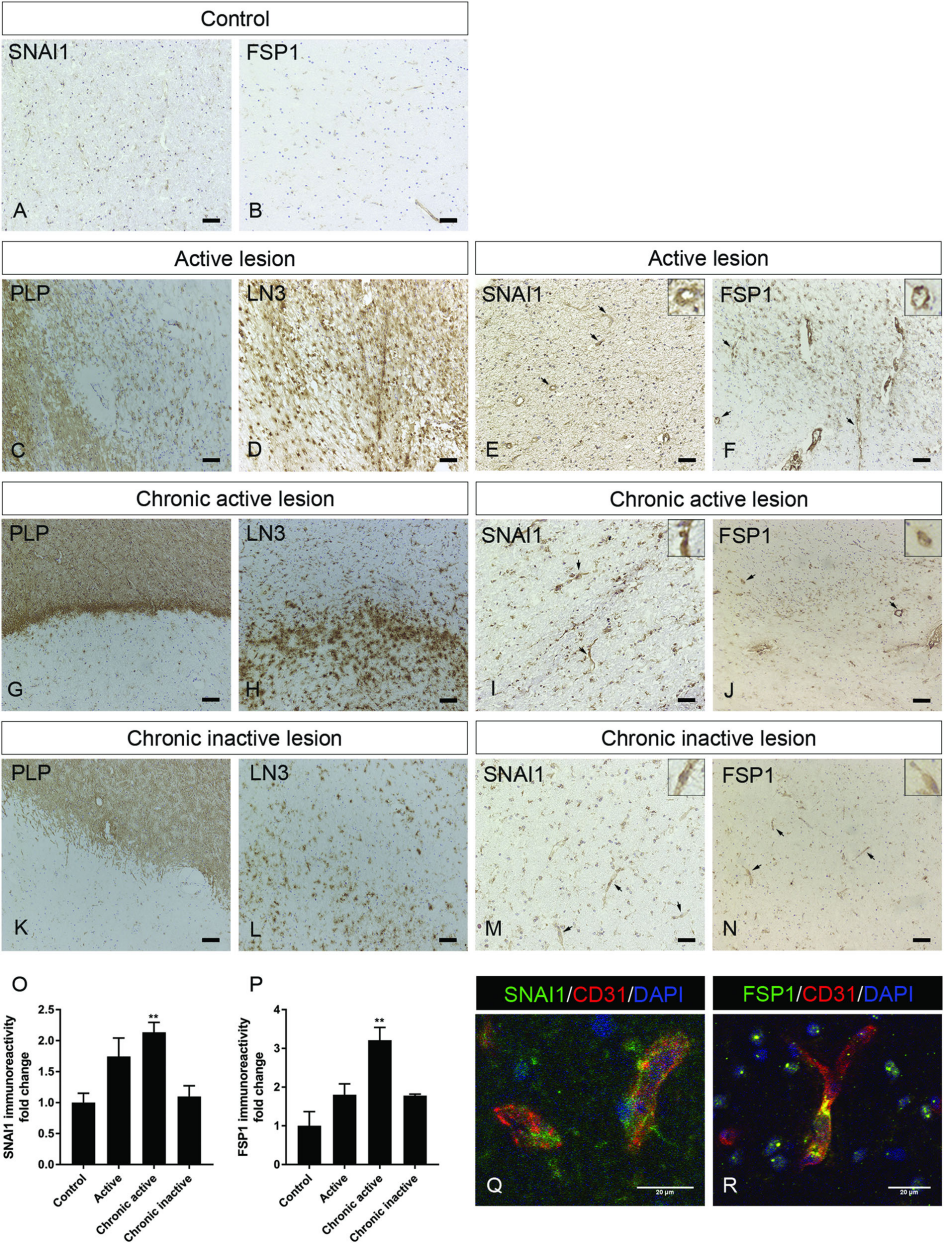
Markers for EndoMT are present in the brain vasculature in MS lesions. **a**, **b** Immunohistochemistry for SNAI1 and FSP1 in non-neurological control brain tissue and active lesion (scale bar 50 µm). **c**–**f** Immunohistochemistry for myelin (PLP) and HLA-DR (LN3) showing an active lesion with increased expression of SNAI1 and FSP1 (scale bar 100 µm). Arrows indicate positive blood vessels. **g**–**j** Immunohistochemistry for myelin (PLP) and HLA-DR (LN3) showing a chronic active lesion with increased expression of SNAI1 and FSP1 (scale bar 100 µm). Arrows indicate positive blood vessels. **k**–**n** SNAI1 and FSP1 expression in chronic inactive lesion, characterized by myelin (PLP) and HLA-DR (LN3) staining (scale bar 100 µm). Arrows indicate positive blood vessels. **o**, **p** SNAI1 and FSP1 staining intensity quantification. **q** Immunofluorescent staining showing co-localization of SNAI1 (green) and CD31 (red) in chronic active lesion (scale bar 20 µm). **r** Immunofluorescent staining showing co-localization of FSP1 (green) and CD31 (red) in chronic active lesion (scale bar 20 µm)

Importantly, TGF-β1 and IL-1β immune-positive astrocytes and macrophages/microglia were found to be present during active and chronic active lesions, highlighting their role during the disease development^48^. Therefore, to determine whether EndoMT features are present in the brain vasculature of human MS patients, we first assessed the expression levels and cellular localization of SNAI1 and FSP1, as markers of EndoMT, in well-characterized WM MS lesions and healthy controls (Table 1). Immunohistochemical analysis of WM from non-neurological controls showed weak vascular SNAI1 and FSP1 protein expression (Fig. 5a, b), whereas both proteins were strongly expressed in MS patients (Fig. 5c–n), with significantly increased expression levels in chronic active lesions (Fig. 5o, p). Particularly, SNAI1 and FSP1 vessel expression is shown in Fig. 5q, r. Taken together, we observe and describe for the first time the presence of EndoMT-related feature in MS patient brain vasculature, suggesting that EndoMT might play an important role during MS pathology.

### Discussion

Here we show that EndoMT drives BEC de-differentiation upon neuro-inflammation and mediates inflammation-induced BECs dysfunction. This transition is characterized by an increase in EndoMT-related TFs, a downregulation of brain endothelial markers, and an upregulation of mesenchymal markers accompanied by morphological changes associated with cytoskeleton reorganization, ultimately leading to BBB dysfunction. EndoMT is the result of a concerted action of multiple TFs^22^, which complicates the study of the downstream effects of each individual one. To investigate the specific role of the most upregulated TF, SNAI1, we generated a BEC cell line overexpressing SNAI1. Using this model system, we show that SNAI1 in itself is sufficient to induce BEC de-differentiation and promote BBB dysfunction. Notably, the higher BBB impairment observed upon TGF-β1 and IL-1β might be the result of the action of the other EndoMT-associated TFs, which were not altered in the specific SNAI1 overexpressing BECs. Furthermore, we provide data supporting the role of TAK1 as master kinase and hub in a network of different pro-inflammatory and EndoMT-inducing signaling pathways and TFs, that include TGF-β1, IL-1β, NF-kB, and SNAI1. Importantly, TAK1 inhibition resulted in reduced transcriptional activity of NF-kB, and in reduced nuclear expression of SNAI1, consequently inhibiting EndoMT induction upon TGF-β1 and IL-1β stimulation, thereby restoring barrier function. Finally, we show increased expression of SNAI1 and FSP1, two well-known EndoMT markers^14,40,49^, in the brain vasculature of MS patients compared to healthy control subjects. Increased SNAI1 and FSP1 expression was observed mostly in chronic active lesions compared to active and chronic inactive lesions^50–52^. Interestingly, EndoMT has been shown to be responsible for endothelial cell de-differentiation in endothelial cells from different vascular beds^16,49,50,53–55^ and has recently raised significant interest in the fields of BECs dysfunction and brain pathologies^19–21,49,56^. However, to date, a putative link between EndoMT and inflammation-induced BBB dysfunction, as seen in MS, remained unknown. Our results, together with previous studies showing tight junctions abnormalities and BBB dysfunction in MS lesions^57–61^, support our hypothesis regarding the occurrence of EndoMT in MS pathology. Importantly, astrocyte and microglia activation is a key process during MS as these glial cells contribute to brain endothelial inflammation and dysfunction via secretion of pro-inflammatory mediators, including TGF-β1 and IL-1β^36–39,62^. Notably, TGF-β1 and IL-1β immune-positive astrocytes and macrophages/microglia were found to be present in active and chronic active lesions, highlighting their role during disease development^48^. Inflammation is one of the major inducers of EndoMT, with TGF-β playing a dominant role in the process^17,40,63^ and IL-1β being able to further enhance its effect^41,64^. Interestingly, both TGF-β and IL-1β downstream signaling cascades have been shown to converge at the level of TAK1, a central regulator of the transcription factors NF-kB as well as AP-1^43,65^. TAK1 belongs to the mitogen-activated protein kinase 3 (MAP3K) kinase family and is involved in the control of TFs like SNAI1, thereby influencing the process of EndoMT^66,67^. Our in vitro data pointing towards a central role of TAK1 signaling in the induction of EndoMT are in line with previous studies reporting that NF-kB activation in BECs is associated with increased BBB permeability^36,68–70^ and that NF-kB inhibition through suppression of TAK1 inhibits epithelial and endothelial de-differentiation^44,71–73^. Nevertheless, other groups proposed a protective role for TAK1 and NF-kB signaling in BBB function, showing that NF-kB activation stabilized the TJ protein occludin and that blocking TAK1 resulted in BBB disruption and BECs death^64^. This apparent contrast might be due to the fact that TAK1 and NF-kB signaling was studied in mice under basal conditions, whereas we demonstrate EndoMT alterations in human BECs upon inflammatory insults. Our data, supporting a role of SNAI1 and EndoMT as underlying mechanism of BBB dysfunction, are in accordance with a previous report where increased SNAI1 expression was found to mediate BBB disruption and bacterial penetration upon meningeal bacterial infection^19^. To date, different downstream pathways involved in CNS EndoMT have been described, including the MEK1/2-ERK1/2 pathway^19^, the MEK5‐ERK5 cascade that in turn upregulates Kruppel-like factor 4 (KLF4)^74^, and the Smad-dependent TGF-β signaling pathway^20,21,74^. Due to the different conditions used in these studies, and due to the multiple signaling pathways and downstream molecules involved in EndoMT, it is challenging to dissect the specific disease-associated signals leading to EndoMT. Our current data do not exclude possible contributions of TAK-1 independent signaling pathways to the observed induction of *SNAI1* mRNA expression and subsequent EndoMT induction. Nevertheless, the inhibition of TAK1 resulted in a significant prevention of features of EndoMT and reinstatement of the BBB function, thus highlighting the key role of TAK1 in the regulation of SNAI1 and EndoMT, in the specific context of BEC neuro-inflammation during MS. Importantly, the therapeutic potential of TAK1 inhibition has been recently explored and it has been shown that blocking TAK1 confers neuroprotection against early brain injury^75^, cerebral ischemia^76^ and experimental autoimmune encephalomyelitis^77^. Although our data show EndoMT-feature during MS pathophysiology, it still remains challenging to determine the exact frequency and location of these events in vivo. Further research will aid in better understanding and characterizing the role of EndoMT during MS pathogenesis and progression, and may provide new leads in the development of specific disease-modifying therapies that directly target the BBB. In conclusion, our data describe a novel mechanism underlying BEC de-differentiation upon neuro-inflammation, pointing to a protective role of OZ (as a TAK1 inhibitor), highlighting its therapeutic potential to attenuate inflammatory events affecting BECs function. Furthermore, with the present study we have broadened the current framework by identifying for the first time EndoMT-associated features in MS.

## Supplementary information

Supplementary figure 1

Supplementary figure 2

Supplementary figure 3

Supplementary figure 4

Supplementary Table 1

Supplementary figure legends

## References

[1] Weiss N, Miller F, Cazaubon S, Couraud PO (2009). The blood-brain barrier in brain homeostasis and neurological diseases. Biochim. Et. Biophys. Acta.

[2] Abbott NJ, Ronnback L, Hansson E (2006). Astrocyte-endothelial interactions at the blood-brain barrier. Nat. Rev. Neurosci..

[3] Tietz S, Engelhardt B (2015). Brain barriers: crosstalk between complex tight junctions and adherens junctions. J. Cell. Biol..

[4] Lassmann H, Van Horssen J, Mahad D (2012). Progressive multiple sclerosis: pathology and pathogenesis. Nat. Rev. Neurol..

[5] Noseworthy JH, Lucchinetti C, Rodriguez M, Weinshenker BG (2000). Multiple Sclerosis. New Engl. J. Med..

[6] Michinaga S, Koyama Y (2017). Protection of the blood–brain barrier as a therapeutic strategy for brain damage. Biol. Pharm. Bull..

[7] Minagar A, Alexander JS (2003). Blood-brain barrier disruption in multiple sclerosis. Mult. Scler..

[8] Reijerkerk A (2013). MicroRNAs regulate human brain endothelial cell-barrier function in inflammation: implications for multiple sclerosis. J. Neurosci..

[9] W Kamphuis W, Derada Troletti C, Reijerkerk A, A Romero I, E de Vries H (2015). The blood-brain barrier in multiple sclerosis: microRNAs as key regulators. CNS Neurol. Disord. Drug Targets.

[10] Engelhardt B, Liebner S (2014). Novel insights into the development and maintenance of the blood–brain barrier. Cell Tissue Res..

[11] Umans RA (2017). CNS angiogenesis and barriergenesis occur simultaneously. Dev. Biol..

[12] Liebner S (2008). Wnt/β-catenin signaling controls development of the blood-brain barrier. J. Cell. Biol..

[13] Obermeier B, Daneman R, Ransohoff RM (2013). Development, maintenance and disruption of the blood-brain barrier. Nat. Med..

[14] Dejana E, Hirschi KK, Simons M (2017). The molecular basis of endothelial cell plasticity. Nat. Commun..

[15] Medici D (2010). Conversion of vascular endothelial cells into multipotent stem-like cells. Nat. Med..

[16] Zeisberg EM (2007). Endothelial-to-mesenchymal transition contributes to cardiac fibrosis. Nat. Med..

[17] Kalluri R, Weinberg RA (2009). The basics of epithelial-mesenchymal transition. J. Clin. Invest..

[18] Armstrong EJ, Bischoff J (2004). Heart valve development: endothelial cell signaling and differentiation. Circ. Res..

[19] Kim BJ (2015). Bacterial induction of Snail1 contributes to blood-brain barrier disruption. J. Clin. Invest..

[20] Krizbai IA (2015). Endothelial-mesenchymal transition of brain endothelial cells: possible role during metastatic extravasation. PLoS One.

[21] Maddaluno L (2013). EndMT contributes to the onset and progression of cerebral cavernous malformations. Nature.

[22] Derada Troletti C, de Goede P, Kamermans A, de Vries HE (2016). Molecular alterations of the blood–brain barrier under inflammatory conditions: the role of endothelial to mesenchymal transition. Biochim. Et. Biophys. Acta.

[23] Arciniegas E, Carrillo LM, De Sanctis JB, Candelle D (2008). Possible role of NFĸB in the embryonic vascular remodeling and the endothelial-mesenchymal transition process. Cell Adhes. Migr..

[24] Lee JG, Kay EP (2012). NF-κB is the transcription factor for FGF-2 that causes endothelial mesenchymal transformation in cornea. Invest. Ophthalmol. Vis. Sci..

[25] Mahler GJ, Farrar EJ, Butcher JT (2013). Inflammatory cytokines promote mesenchymal transformation in embryonic and adult valve endothelial cells. Arterioscler. Thromb. Vasc. Biol..

[26] Lamouille S, Xu J, Derynck R (2014). Molecular mechanisms of epithelial–mesenchymal transition. Nat. Rev. Mol. Cell Biol..

[27] Weksler B (2005). Blood-brain barrier-specific properties of a human adult brain endothelial cell line. Faseb J..

[28] Ninomiya-Tsuji J (2003). A resorcylic acid lactone, 5Z-7-oxozeaenol, prevents inflammation by inhibiting the catalytic activity of TAK1 MAPK kinase kinase. J. Biol. Chem..

[29] Alvarez JI, Cayrol R, Prat A (2011). Disruption of central nervous system barriers in multiple sclerosis. Biochim. Et. Biophys. Acta.

[30] Cayrol R (2008). Activated leukocyte cell adhesion molecule promotes leukocyte trafficking into the central nervous system. Nat. Immunol..

[31] Ifergan I (2006). Statins reduce human blood–brain barrier permeability and restrict leukocyte migration: relevance to multiple sclerosis. Ann. Neurol..

[32] Kebir H (2007). Human T H 17 lymphocytes promote blood-brain barrier disruption and central nervous system inflammation. Nat. Med..

[33] Dull T (1998). A third-generation lentivirus vector with a conditional packaging system. J. Virol..

[34] Giaever I, Keese CR (1991). Micromotion of mammalian cells measured electrically. Proc. Natl Acad. Sci..

[35] Grada A, Otero-Vinas M, Prieto-Castrillo F, Obagi Z, Falanga V (2017). Research techniques made simple: analysis of collective cell migration using the wound healing assay. J. Invest. Dermatol..

[36] Didier N (2003). Secretion of interleukin‐1β by astrocytes mediates endothelin‐1 and tumour necrosis factor‐α effects on human brain microvascular endothelial cell permeability. J. Neurochem..

[37] Moynagh PN (2005). The interleukin‐1 signalling pathway in astrocytes: a key contributor to inflammation in the brain. J. Anat..

[38] Rothwell NJ (1999). Cytokines‐killers in the brain?. J. Physiol..

[39] Wyss-Coray T, Borrow P, Brooker MJ, Mucke L (1997). Astroglial overproduction of TGF-β1 enhances inflammatory central nervous system disease in transgenic mice. J. Neuroimmunol..

[40] Medici D, Potenta S, Kalluri R (2011). Transforming growth factor-β2 promotes Snail-mediated endothelial–mesenchymal transition through convergence of Smad-dependent and Smad-independent signalling. Biochem. J..

[41] Maleszewska M (2013). IL-1β and TGFβ2 synergistically induce endothelial to mesenchymal transition in an NFκB-dependent manner. Immunobiology.

[42] Mihaly S, Ninomiya-Tsuji J, Morioka S (2014). TAK1 control of cell death. Cell Death Differ..

[43] Sakurai H (2012). Targeting of TAK1 in inflammatory disorders and cancer. Trends Pharmacol. Sci..

[44] Lee ES, Boldo LS, Fernandez BO, Feelisch M, Harmsen MC (2017). Suppression of TAK1 pathway by shear stress counteracts the inflammatory endothelial cell phenotype induced by oxidative stress and TGF-β1. Sci. Rep..

[45] Scholz R (2010). Autoactivation of transforming growth factor β-activated kinase 1 is a sequential bimolecular process. J. Biol. Chem..

[46] Singhirunnusorn P, Suzuki S, Kawasaki N, Saiki I, Sakurai H (2005). Critical roles of threonine 187 phosphorylation in cellular stress-induced rapid and transient activation of transforming growth factor-β-activated kinase 1 (TAK1) in a signaling complex containing TAK1-binding protein TAB1 and TAB2. J. Biol. Chem..

[47] Lawrence T (2009). The nuclear factor NF-κB pathway in inflammation. Cold Spring Harb. Perspect. Biol..

[48] De Groot CJ, Montagne L, Barten AD, Sminia P, Van Der Valk P (1999). Expression of transforming growth factor (TGF)-β1,-β2, and-β3 isoforms and TGF-β type I and type II receptors in multiple sclerosis lesions and human adult astrocyte cultures. J. Neuropathol. Exp. Neurol..

[49] Evrard SM (2016). Endothelial to mesenchymal transition is common in atherosclerotic lesions and is associated with plaque instability. Nat. Commun..

[50] Cao Y, Feng B, Chen S, Chu Y, Chakrabarti S (2014). Mechanisms of endothelial to mesenchymal transition in the retina in diabetes. Invest. Ophthalmol. Vis. Sci..

[51] Lassmann H, van Horssen J (2011). The molecular basis of neurodegeneration in multiple sclerosis. FEBS Lett..

[52] Van der Valk P, De Groot CJA (2000). Staging of multiple sclerosis (MS) lesions: pathology of the time frame of MS. Neuropathol. Appl. Neurobiol..

[53] Mahmoud MM (2017). Shear stress induces endothelial-to-mesenchymal transition via the transcription factor Snail. Sci. Rep..

[54] Ribera J (2017). A small population of liver endothelial cells undergoes endothelial-to-mesenchymal transition in response to chronic liver injury. Am. J. Physiol. Gastrointest. Liver Physiol..

[55] Xiao L (2015). Tumor endothelial cells with distinct patterns of TGFβ-driven endothelial-to-mesenchymal transition. Cancer Res..

[56] Bai Y (2018). Circular RNA DLGAP4 ameliorates ischemic stroke outcomes by targeting miR-143 to regulate endothelial-mesenchymal transition associated with blood–brain barrier integrity. J. Neurosci..

[57] Alvarez JI (2015). Focal disturbances in the blood–brain barrier are associated with formation of neuroinflammatory lesions. Neurobiol. Dis..

[58] Kirk J, Plumb J, Mirakhur M, McQuaid S (2003). Tight junctional abnormality in multiple sclerosis white matter affects all calibres of vessel and is associated with blood-brain barrier leakage and active demyelination. J. Pathol..

[59] Kwon EE, Prineas JW (1994). Blood-brain barrier abnormalities in longstanding multiple sclerosis lesions. An immunohistochemical study. J. Neuropathol. Exp. Neurol..

[60] Plumb J, McQuaid S, Mirakhur M, Kirk J (2002). Abnormal endothelial tight junctions in active lesions and normal-appearing white matter in multiple sclerosis. Brain Pathol..

[61] Vos CM (2005). Blood–brain barrier alterations in both focal and diffuse abnormalities on postmortem MRI in multiple sclerosis. Neurobiol. Dis..

[62] Burm SM (2016). Expression of IL-1β in rhesus EAE and MS lesions is mainly induced in the CNS itself. J. Neuroinflamm..

[63] Cooley BC (2014). TGF-β signaling mediates endothelial-to-mesenchymal transition (EndMT) during vein graft remodeling. Sci. Transl. Med..

[64] Rieder F (2011). Inflammation-induced endothelial-to-mesenchymal transition: a novel mechanism of intestinal fibrosis. Am. J. Pathol..

[65] Dey N, Liu T, Garofalo RP, Casola A (2011). TAK1 regulates NF-κB and AP-1 activation in airway epithelial cells following RSV infection. Virology.

[66] Landström M (2010). The TAK1–TRAF6 signalling pathway. Int. J. Biochem. Cell. Biol..

[67] Souihol, C. E., Harmsen, M. C., Evans, P. C. & Krenning, G. Endothelial-mesenchymal transition in atherosclerosis. *Cardiovasc. Res.* **114**, 565–577 (2018). 10.1093/cvr/cvx253 29309526

[68] Abbott NJ, Patabendige AA, Dolman DE, Yusof SR, Begley DJ (2010). Structure and function of the blood–brain barrier. Neurobiol. Dis..

[69] Alvarez JI, Katayama T, Prat A (2013). Glial influence on the blood brain barrier. Glia.

[70] Coelho-Santos V (2015). The TNF-α/Nf-κ B signaling pathway has a key role in methamphetamine-induced blood–brain barrier dysfunction. J. Cereb. Blood Flow Metab..

[71] Dvashi Z, Goldberg M, Adir O, Shapira M, Pollack A (2015). TGF-β1 induced transdifferentiation of rpe cells is mediated by TAK1. PLoS One.

[72] Gardner A (2012). The critical role of TAK1 in accentuated epithelial to mesenchymal transition in obliterative bronchiolitis after lung transplantation. Am. J. Pathol..

[73] Strippoli R (2012). Inhibition of transforming growth factor-activated kinase 1 (TAK1) blocks and reverses epithelial to mesenchymal transition of mesothelial cells. PLoS One.

[74] Cuttano R (2016). KLF4 is a key determinant in the development and progression of cerebral cavernous malformations. EMBO Mol. Med..

[75] Zhang D (2015). TGFβ-activated kinase 1 (TAK1) inhibition by 5Z-7-oxozeaenol attenuates early brain injury after experimental subarachnoid hemorrhage. J. Biol. Chem..

[76] White BJ (2012). Protection from cerebral ischemia by inhibition of TGFβ-activated kinase. Exp. Neurol..

[77] Lu L (2017). Central administration of 5Z-7-oxozeaenol protects experimental autoimmune encephalomyelitis mice by inhibiting microglia activation. Front. Pharmacol..

