# Supplementary figures and images for: Inflammation-induced endothelial to mesenchymal transition promotes brain endothelial cell dysfunction and occurs during multiple sclerosis pathophysiology
Source: Cell Death Dis. 2019 Jan 18;10(2):45. doi: 10.1038/s41419-018-1294-2 (PMC6361981; doi:10.1038/s41419-018-1294-2)

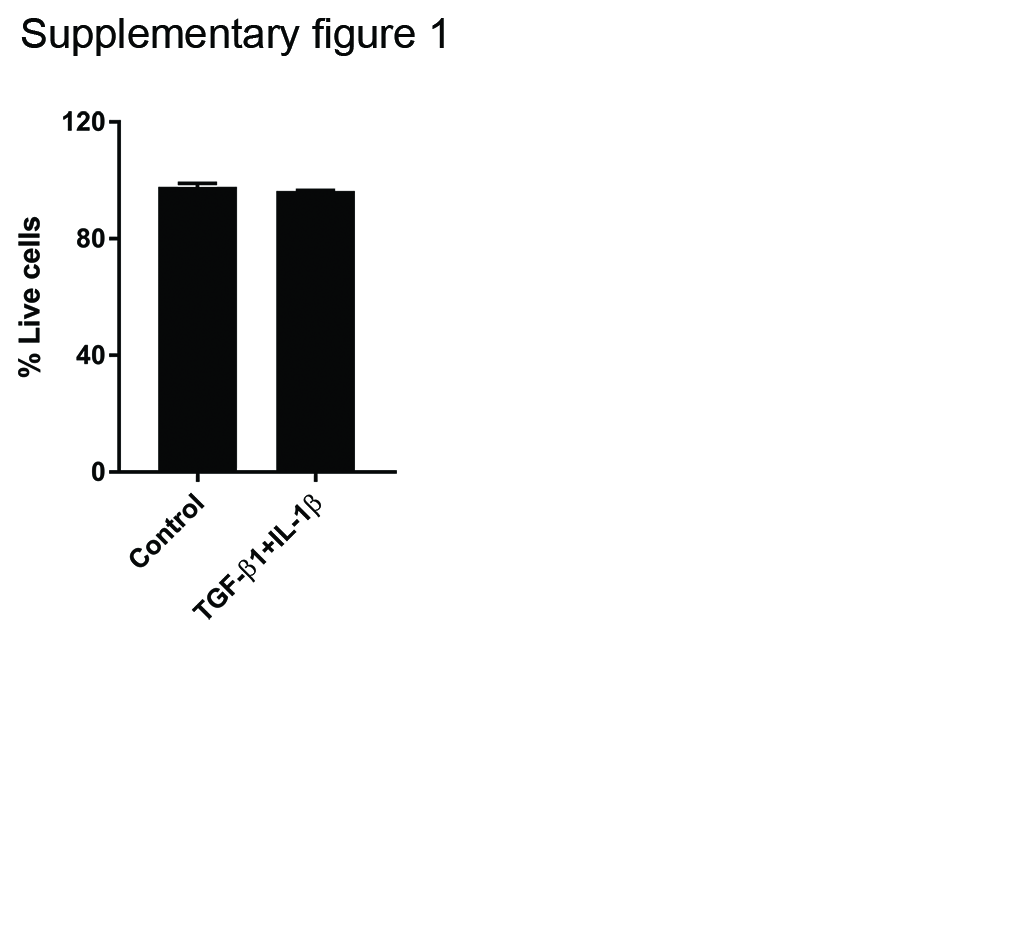

Supplement: Supplementary file 1 — Supplementary figure 1 [file 41419_2018_1294_MOESM1_ESM.png]

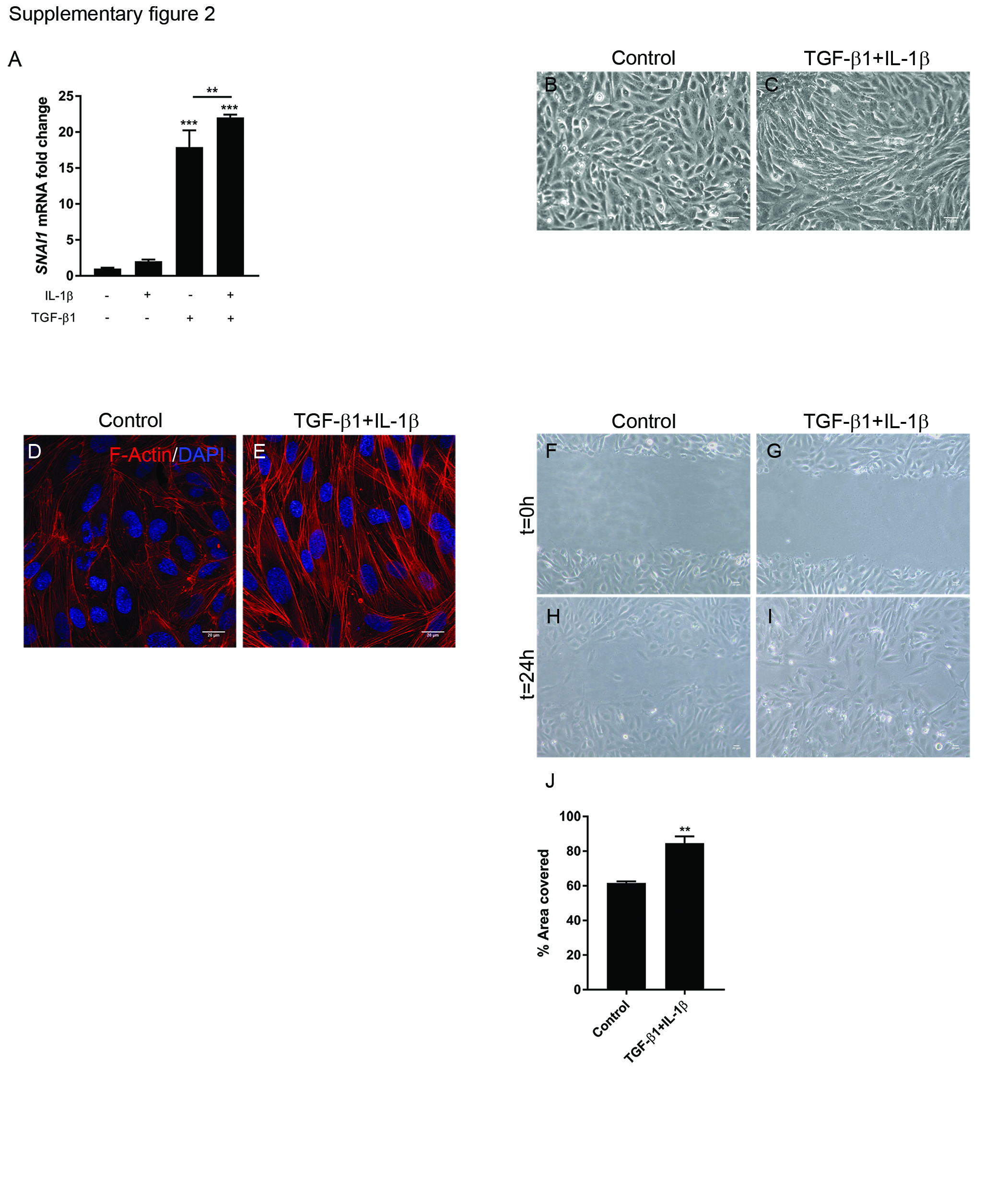

Supplement: Supplementary file 2 — Supplementary figure 2 [file 41419_2018_1294_MOESM2_ESM.png]

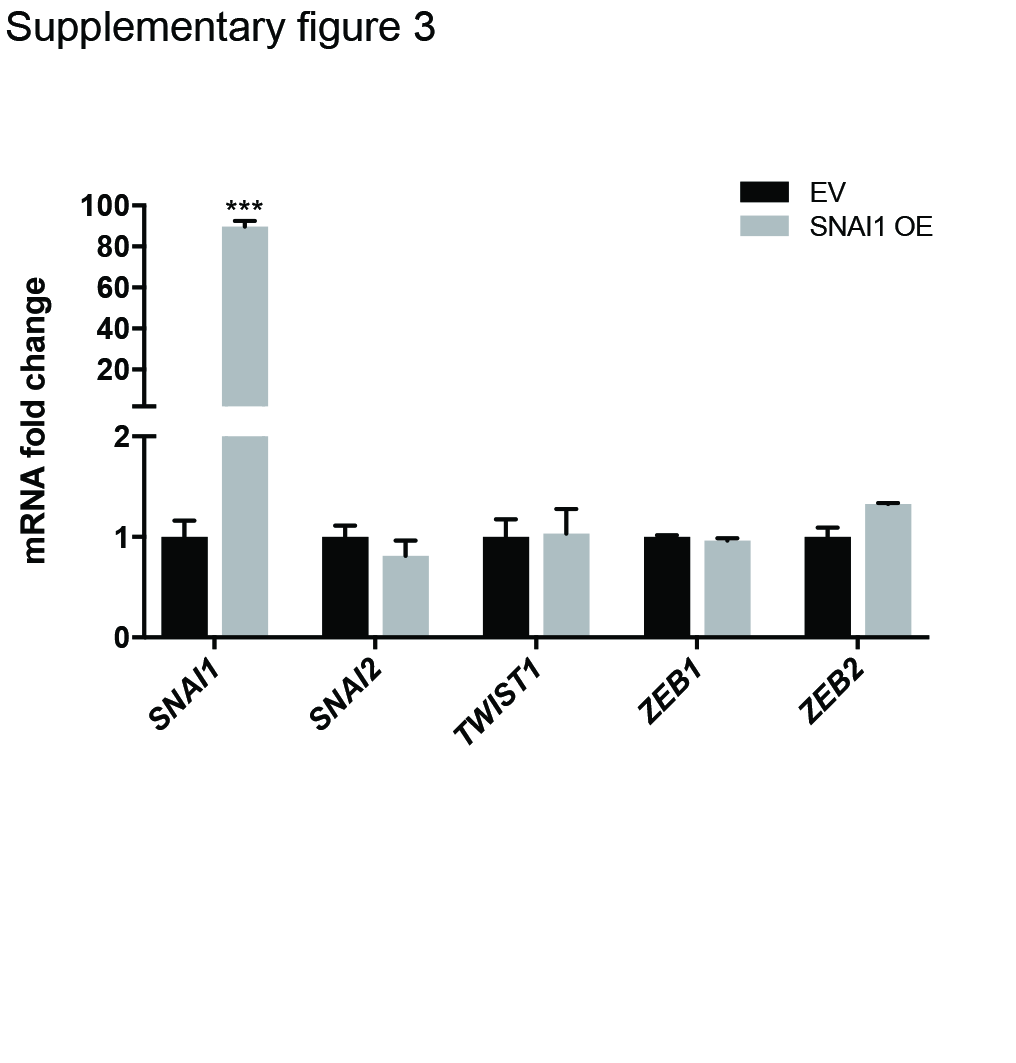

Supplement: Supplementary file 3 — Supplementary figure 3 [file 41419_2018_1294_MOESM3_ESM.png]

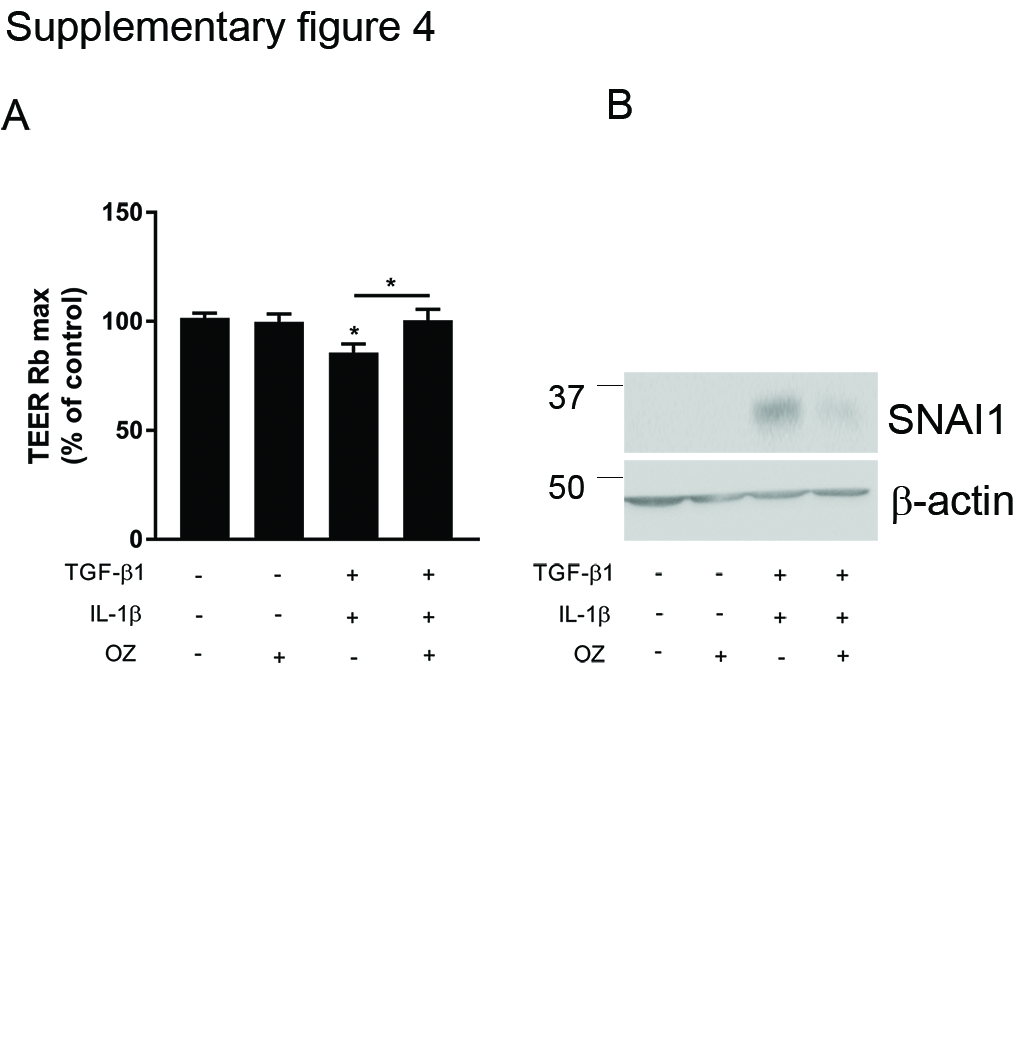

Supplement: Supplementary file 4 — Supplementary figure 4 [file 41419_2018_1294_MOESM4_ESM.png]
