# Supplementary material for: Inflammation-induced endothelial to mesenchymal transition promotes brain endothelial cell dysfunction and occurs during multiple sclerosis pathophysiology
Source: Cell Death Dis. 2019 Jan 18;10(2):45. doi: 10.1038/s41419-018-1294-2 (PMC6361981; doi:10.1038/s41419-018-1294-2)
Supplement: Supplementary file 6 — Supplementary figure legends [file 41419_2018_1294_MOESM6_ESM.docx]

**Supplementary figure 1. TGF-β1 and IL-1β do not affect cell viability**

Cell viability of TGF-β1 and IL-1β-stimulated BECs was determined using exclusion of trypan blue and calculated as the number of viable cells divided by the total number of cells within the grids on the hemocytometer and plotted as % of live cells. Data presented are the mean of triplicate values ± SEM of three independent experiments. Statistical analysis was performed using Student’s t-test.

**Supplementary figure 2. Characterization of EndoMT in inflamed BECs**

**(a)** *SNAI1* mRNA expression levels were determined by qRT-PCR in BECs stimulated with different pro-inflammatory stimuli as indicated in the figure. **(B,C)** Morphological changes upon BECs stimulation with TGF-β1 and IL-1β were observed under the microscope (scale bar 20 µm). **(D,E)** F-actin staining of TGF-β1 and IL-1β-stimulated BECs was performed using phalloidin and imaged under the microscope (scale bar 20 µm). **(F-J)** BECs were cultured to confluency and subsequently a scratch assay was performed as described in the material and methods section (scale bar 20 µm). Data presented are the mean of triplicate values ± SEM of three independent experiments. Statistical analysis was performed using Student’s t-test where ** p<0.01.

**Supplementary figure 3. SNAI1 overexpression does not affect EndoMT-related transcription factors in BEC**

**(A)** *SNAI1*, *SNAI2,* *TWIST1*, *ZEB1* and *ZEB2* mRNA expression levels were measured in SNAI1-overexpressing cells by qRT-PCR. Values were normalized to *GAPDH* and plotted as fold change relative to empty vector-transduced cells. Data presented are the mean of triplicate values ± SEM of three independent experiments. Statistical analysis was performed using Student’s t-test.

**Supplementary figure 4. EndoMT in primary human BECs**

**(A)** The effect of TGF-β1 and IL-1β in the presence or absence of OZ treatment in primary human BECs was assessed by measuring the TEER (Rb, barrier resistance), plotted as % of control BECs. Data presented are the mean of triplicate values ± SEM of three independent experiments. Statistical analysis was performed using one-way ANOVA where * p < 0.05, ** p < 0.01, *** p < 0.001 with post-hoc Bonferroni correction. **(B)** Primary human BECs were stimulated for 24 h as indicated in the figure, cells were lysed and SNAI1 protein content was measured by western blot. Values were normalized to β-actin.

**Supplementary table 1. Primers sequences**

Primers sequences used.
